# Association of antimicrobial perioperative prophylaxis with cefuroxime plus metronidazole or amoxicillin/clavulanic acid and surgical site infections in colorectal surgery

**DOI:** 10.1186/s13756-023-01307-y

**Published:** 2023-09-19

**Authors:** Elisavet Stavropoulou, Andrew Atkinson, Marie-Christine Eisenring, Christoph A. Fux, Jonas Marschall, Laurence Senn, Nicolas Troillet

**Affiliations:** 1 Service of Infectious Diseases, Central Institute, Valais Hospital, Sion, Switzerland; 2 Service of Infectious Diseases, Department of Medicine, Lausanne University Hospital, University of Lausanne, Lausanne, Switzerland; 3 Department of Infectious Diseases, Bern University Hospital, Inselspital, University of Bern, Bern, Switzerland; 4 Swissnoso, National Center for Infection Prevention, Bern, Switzerland; 5 Division of Infectious Diseases and Infection Prevention, Kantonsspital Aarau, Aarau, Switzerland; 6 Division of Infectious Diseases, Washington University School of Medicine, St. Louis, MO USA; 7 Infection Prevention and Control Unit, Service of Infectious Diseases, Lausanne University Hospital, University of Lausanne, Lausanne, Switzerland

**Keywords:** Surgical site infection, Antibiotic prophylaxis, Colorectal surgery, Amoxicillin/clavulanic acid, Cefuroxime, Metronidazole

## Abstract

**Objective:**

To compare intravenous (IV) amoxicillin/clavulanic acid (A/CA) to IV cefuroxime plus metronidazole (C + M) for preventing surgical site infections (SSI) in colorectal surgery.

**Background:**

Given their spectra that include most Enterobacterales and anaerobes, C + M is commonly recommended as prophylaxis of SSI in colorectal surgery. A/CA offers good coverage of Enterobacterales and anaerobes as well, but, in contrast to C + M, it also includes *Enterococcus faecalis* which is also isolated from patients with SSI and could trigger anastomotic leakage.

**Methods:**

Data from a Swiss SSI surveillance program were used to compare SSI rates after class II (clean contaminated) colorectal surgery between patients who received C + M and those who received A/CA. We employed multivariable logistic regression to adjust for potential confounders, along with propensity score matching to adjust for group imbalance.

**Results:**

From 2009 to 2018, 27,922 patients from 127 hospitals were included. SSI was diagnosed in 3132 (11.2%): 278/1835 (15.1%) in those who received A/CA and 2854/26,087 (10.9%) in those who received C + M (*p* < 0.001). The crude OR for SSI in the A/CA group as compared to C + M was 1.45 [CI 95% 1.21–1.75]. The adjusted OR was 1.49 [1.24–1.78]. This finding persisted in a 1:1 propensity score matched cohort of 1835 patients pairs with an OR of 1.60 [1.28–2.00]. Other factors independently associated with SSI were an ASA score > 2, a longer duration of operation, and a reoperation for a non-infectious complication. Protective factors were female sex, older age, antibiotic prophylaxis received 60 to 30 min before surgery, elective operation, and endoscopic approach.

**Conclusions:**

Despite its activity against enterococci, A/CA was less effective than C + M for preventing SSI, suggesting that it should not be a first choice antibiotic prophylaxis for colorectal surgery.

**Supplementary Information:**

The online version contains supplementary material available at 10.1186/s13756-023-01307-y.

## Background

Healthcare associated infections (HAIs) still constitute one of the most frequent complications linked to medical care. In 2011, the World Health Organization (WHO) estimated that 7% of hospitalized patients in developed countries presented at least one HAI during their stay [1]. In the European Union, their yearly burden has been estimated as high as 501 disability-adjusted life years per 100,000 general population [2]. In Switzerland, the prevalence of HAI among hospitalized patients was recently estimated to be 5.9%, with surgical site infections (SSIs) being the most common type representing 29% of all HAI [3].

Guidelines for the prevention of SSIs all recommend perioperative surgical antimicrobial prophylaxis (SAP) with a high level of evidence for non-clean operations and clean operations that include foreign bodies [4, 5]. Colorectal surgery carry the highest risk of SSI with an incidence that can be above 25% [6, 7]. SAP covering the digestive flora is thus particularly relevant for this type of surgery. As preferred options, most guidelines recommend a combination of cefuroxime or cefazolin plus metronidazole (C + M) to cover aerobic and anaerobic bacteria constituting the colorectal flora [8–10]. However, *Enterococcus faecalis*, which may be isolated in up to 46% of patients suffering from SSI after colorectal surgery [6, 11], is intrinsically resistant to cephalosporins. Although the role of enterococci in causing abdominal infections is controversial [12], recent data suggest that they might proliferate after colorectal surgery and predispose to anastomotic leakage which may result in secondary organ/space SSI [13]. Amoxicillin/clavulanic acid (A/CA), an antibiotic used for surgical SAP instead of C + M by some surgeons, is active against *E. faecalis* and offers a similar activity as C + M against most other bacteria of the normal colon flora.

The current study aimed at comparing C + M and A/CA as an SAP for colorectal surgery based on data from the Swiss SSI surveillance program by the National Center for Infection Prevention, [Swissnoso].

## Materials and methods

The Swissnoso SSI surveillance program is described in detail in former publications [7, 14]. Data are collected through chart review by trained infection control nurses who are supervised by infectious diseases physicians; a rigorous post-discharge surveillance is conducted by standardized phone interviews and additional data collection in case of suspicion of SSI. SSIs are diagnosed according to the definitions of the Centers for Diseases Control and Prevention (CDC) and classified as superficial incisional, deep incisional, or organ-space infections [15, 16]. One to three causative microorganisms per SSI can be registered if intraoperative cultures have been obtained. Data are electronically entered into a centralized database. Staff members of the Swissnoso SSI surveillance team periodically perform on-site audits to ascertain data quality, as described elsewhere [7, 17, 18]. The study was approved by the ethical committee of the Canton of Bern (project-ID: 2019-00294).

All patients who underwent colorectal surgery with a contamination class of II (clean contaminated) and a complete follow-up of 30 days between 2009 and 2018 were included if they were older than 16, not already on antibiotics, and had received either C + M or A/CA for prophylaxis. Reliable data about doses and re-dosing intervals not being available, we assumed that the Swiss guidelines which recommend 1.5 g for cefuroxime (re-dosing after 3–4 h), 500 mg for metronidazole (re-dosing after 8 h), and 2.2 g for A/AC (re-dosing after 2 h) had been respected [10].

SSI rates were compared between the comparator groups using the Kruskal–Wallis test and the chi-square test as appropriate. Uni- and multivariable logistic regression models were fitted with SSI as a dependent variable and the following independent co-variables: A/AC versus C + M, age, sex, American Society of Anesthesiologists (ASA) score, type of procedure (colon or rectal), presence of colorectal cancer, endoscopic approach versus laparotomy, emergent vs. elective procedure, operation duration, time from antimicrobial prophylaxis administration to incision (specifically, administration of the first antibiotic if multiple were given), and re-operation for a non-infectious complication during the follow-up period.

Variables with a *p* value ≤ 0.1 in univariable models were fitted in an adjusted model using forwards, then backwards selection. Sandwich-type standard errors were calculated to further adjust for intra-hospital correlation.

A 1:1 propensity score analysis was conducted on patient pairs matched for age, sex, ASA score, operation duration, timing of SAP, elective procedure, laparoscopic procedure, hospital size, and re-operation.

All tests were two-tailed. *p* values < 0.05 were considered statistically significant. Statistical analyses were performed with R, version 3.6.1 [19].

## Results

The analyses included 27,922 patients from 127 hospitals: 26,087 (93%) from 123 hospitals in the C + M group and 1835 (7%) from 99 hospitals in the A/CA group (see Additional file 1). The latter were paired with 1835 patients in the C + M group for propensity score analysis. Table 1 summarizes patient and surgery characteristics.

**Table 1:** Patients’ and operations’ characteristics by antibiotic group

Characteristics	Cefuroxime + metronidazole (N = 26,087)	Amoxicillin/clavulanic acid (N = 1835)	*p* Value
Age, median (IQR)	68 [58, 77]	69 [59, 78]	0.03
*Sex, N *(*%*)			
Male	13,048 (50)	942 (51.3)	0.3
Female	13,039 (50)	893 (48.7)	
ASA score > 2, N (%)	9254 (35.5)	650 (35.4)	0.9
Rectal procedures, N (%)	1654 (6.3)	134 (7.3)	0.1
Elective procedures, N (%)	22,946 (88.0)	1595 (86.9)	0.2
Laparoscopic surgery, N (%)	13,975 (53.6)	912 (49.7)	0.001
Reoperation for a non-infectious complication, N (%)	1376 (5.3)	100 (5.4)	0.8
Operation duration in minutes, median (IQR)	169 [122, 227]	162 [117, 229]	0.001
Timing of antibiotic prophylaxis in minutes, median (IQR)	− 35 [− 50, − 20]	− 36 [− 55, − 20]	0.001
Operation duration > 75th percentile, N (%)	11,361 (43.6)	746 (40.7)	0.02
*Operations by hospital size, N *(*%*)			
< 200 beds	10,579 (40.6)	649 (35.4)	< 0.001
200–499 beds	10,447 (40.0)	1063 (57.9)	
≥ 500 beds	5061 (19.4)	123 (6.7)	

*The inclusion of colon surgery in the surveillance system is mandatory. Hospitals must choose at least 2 other operations from a catalogue of 13 that includes rectal surgery

More laparoscopic operations were performed in the C + M group compared to the A/CA group (53.6% vs. 49.7%, *p* = 0.001) and patients in the C + M group were more likely to have longer procedures (43.6% vs. 40.7% exceeding the 75th percentile, *p* = 0.02). Patients in the C + M group were more likely to be hospitalized in small (< 200 beds) and large (> 500 beds) hospitals while patients in the A/CA group were more likely to be hospitalized in medium sized hospitals (200–500 beds), *p* < 0.001.

Table 2 shows the overall SSI rates in both groups as well as specific rates for superficial incisional, deep incisional, and organ/space infections.

**Table 2:** Surgical site infections (SSI) rates by antibiotic group

	Cefuroxime + metronidazole (N = 26,087)	Amoxicillin/clavulanic acid (N = 1835)	*p* Value
Overall, N (%)	2854 (10.9)	278 (15.1)	< 0.001
Superficial incisional, N (%)	853 (3.3)	108 (5.9)	< 0.001*
Deep incisional, N (%)	331 (1.3)	32 (1.7)	0.1*
Organ space, N (%)	1670 (6.4)	138 (7.5)	0.07*

*Not corrected for multiple testing

A/CA was associated with a significantly increased risk for SSI in the crude and adjusted multilevel models (adjusted odds ratio [aOR] 1.49; 95% CI 1.24–1.78; *p* < 0.001). An ASA score > 2, an operation duration > the 75th percentile and a re-operation for a non-infectious complication were other co-variables independently associated with a higher SSI risk, while female sex, older age, administration of SAP one hour to 30 min before incision, elective surgery and laparoscopic procedures were associated with a decreased SSI risk (Table 3).

**Table 3:** Risk factors for SSI

Risk factor	Crude odds ratio [95% CI]	*P* value	Adjusted odds ratio [95% CI]	*p* Value
Amoxicillin/clavulanic acid	1.45 [1.21, 1.75]	< 0.001	1.49 [1.24, 1.78]	< 0.001
Age (10 year steps)	1.03 [1.00, 1.06]	0.05	0.96 [0.93, 0.98]	0.003
Female sex	0.67 [0.62, 0.73]	< 0.001	0.75 [0.69, 0.81]	< 0.001
ASA score > 2	1.67 [1.57, 1.79]	< 0.001	1.44 [1.33, 1.56]	< 0.001
Operation duration (per 30 min)	1.08 [1.06, 1.10]	< 0.001	1.08 [1.06, 1.09]	< 0.001
Antibiotic prophylaxis (in 30 min steps prior to incision)*	0.94 [0.89, 0.99]	0.03	0.95 [0.91, 0.99]	0.02
Duration of surgery > 75th percentile	1.38 [1.24, 1.53]	< 0.001	ns	
Elective surgery	0.59 [0.52, 0.66]	< 0.001	0.75 [0.66, 0.85]	< 0.001
Laparoscopic interventions	0.53 [0.47, 0.59]	< 0.001	0.60 [0.54, 0.67]	< 0.001
Rectal surgery	1.09 [0.75, 1.57]	0.7	ns	
*Hospital size *(*N beds*)				
< 200	1 (reference)			
200–499	1.12 [0.85, 1.46]	0.43	ns	
500+	1.30 [1.07, 1.58]	0.009	ns	
Re-operation**	4.85 [3.82, 6.15]	< 0.001	4.31 [3.37, 5.52]	< 0.001

*Of the first antibiotic administered (if in combination)

**For a non-infectious complication during the follow-up period

CI, confidence interval; ASA, American Society of Anesthesiology; ns, not significant (not retained in the multivariable model)

Baseline characteristics of the comparator groups used for the propensity score analysis were similar with the exceptions of longer operations (> 75th percentile) (36% in C + M vs. 40.7% in A/CA, *p* = 0.003); rectal procedures (4% vs. 7.3%, *p* < 0.001) and hospital size (< 200 beds: 46.3% vs. 35.4%, *p* < 0.001). It revealed crude SSI rates of 10.1% in the C + M group and 15.1% in the A/CA group (*p* < 0.001) and an aOR for A/CA of 1.60 [1.28–2.00], *p* < 0.001.

Specific results of univariate and multivariable analyses for superficial incisional, deep incisional and organ-space SSIs are presented in supplementary material (Additional file 1: Tables A, B, C). The OR and aOR of A/AC for organ-space SSI were 1.23 [0.99–1.53] (*p* = 0.06) and 1.27 [1.01–1.60] (*p* = 0.05), respectively.

Microbiological results were available for 1574/2854 (55.2%) patients with SSI in the C + M group and for 184/278 (66.2%) in the A/CA group. *Enterococcus faecalis* grew in 113/1574 (7.2%) cultures after C + M and 8/184 (4.3%) after A/CA (*p* = 0.15). *E. faecium* grew in 119/1574 (7.5%) cultures after C + M and 2/184 (1.1%) after A/AC (*p* = 0.001).

## Discussion

The colorectal SSI rate of 11.2% seen in this cohort of 27,992 patients of over a ten-year period is comparable to findings reported from other studies [6, 7, 20–22]. Such high rates of infectious complications represent a substantial burden for patients, hospitals and society [2] that should be decreased by well-implemented evidence-based preventive measures. In this attempt, SAP constitutes a major step. It aims at establishing a bactericidal concentration of the agent in the serum and tissues when the incision is made and requires the selection of an antibiotic whose spectrum covers the flora expected to be present at the surgical site [4, 5]. Most guidelines recommend a combination of a cephalosporin and metronidazole or an extended spectrum penicillin as first choices in colorectal surgery [8, 10].

In Switzerland, cefuroxime + metronidazole (C + M) is much more widely used than amoxicillin/clavulanic acid (A/CA). In contrast to C + M, A/CA is active against most enterococci (particularly *E. faecalis*), which constitute an important part of the gut flora and may contribute to the development of anastomotic leakage, thus leading to surgical site infection after colorectal surgery [13]. This, in addition to its lower cost and being considered an “access” antimicrobial in the World Health Organisation AWaRe (Access, Watch, Reserve) classification [23], could motivate the use of A/CA instead of C + M for perioperative antibiotic prophylaxis in colorectal surgery.

Despite these considerations, A/CA was not better in preventing colorectal SSI as C + M, even for intraperitoneal (organ /space) infections that anastomotic leakage is presumed to cause according to the findings of an experimental study [13]. We found no significant difference in *E. faecalis* rates between the two prophylaxis regimens suggesting that a single dose of A/AC is not sufficient to significantly decrease *E. faecalis* inoculum.Surprisingly, *E. faecium*, which is frequently resistant to amoxicillin (76 to 78% resistance rate from 2009 to 2018 in Switzerland according to ANRESIS, (www.anresis.ch), and always resistant to cefuroxime, grew in fewer intraoperative cultures when SSIs occurred after A/CA than after C + M. Beside the possible lack of efficacy of a single dose of A/CA, this may simply have occurred by chance. Even though enterococci may be detected in intraoperative cultures when managing an SSI, our data did not suggest this was a common scenario. Also, the pathogenic value of enterococci is not always clear and they may only be colonizing bystanders. The better performance of C + M may be explained by a higher coverage of Enterobacterales and anaerobes. This may be particularly true for *Escherichia coli*, an important cause of SSIs after colorectal surgery. Indeed, despite the increasing rates of resistance of *E. coli* to 2nd generation cephalosporins (2GC) due to extended-spectrum beta-lactamases, cefuroxime remains more active than A/CA, with resistance rates of 6 to 14% for 2GC and 10 to 24% for A/CA during the study period in Switzerland (www.anresis.ch). Similarly, anaerobes, particularly *Bacteroides* spp., show higher susceptibility to metronidazole [23].

Remarkably, A/CA performed significantly worse only in superficial infections which may be partly caused by representatives of the skin flora. These microorganisms would nevertheless be equally well covered by both regimens. Thus, their possible presence cannot explain the higher effectiveness of C + M which might therefore also be due to a better coverage of Enterobacteriales and anaerobes as well as pharmacokinetic (tissue concentrations) or pharmacodynamic parameters (bactericidal effects).

Other studies also found a higher risk for SSI when comparing prophylaxis with A/CA to a cephalosporin-based regimen in digestive surgery. However, in contrast to our findings, these differences did not reach statistical significance [24–26].

As in other studies [27–34], we identified a high ASA score, male sex, long duration of operation, and re-operation for a non-infectious complication during the follow-up period as risk factors for SSI, while elective operations, laparoscopic interventions and the correct timing of SAP were protective factors. Surprisingly, an older age, as measured in 10-year steps, appeared protective (Table 3).

Of note, although there appears to be some differences between the investigated groups (e.g. timing of prophylaxis, operation duration, age) according to the statistical testing framework, these differences may reflect the large number of interventions studied (and consequently high statistical power to detect differences) and not be of clinically relevant.

Our study has some limitations. As in every observational study, unknown or unrecognized confounders may have influenced its findings. Nonetheless, the size of the cohort, its limitation to class II (clean contaminated) operations, the completeness of the data, the multivariable analyses that included many potential confounders, and the propensity score analysis all argue for the robustness of our findings.

Second, no data were available on the administration of perioperative oral antibiotics and mechanical bowel preparation, which can further prevent SSI [35, 36] and could have been unevenly distributed between patients who received C + M versus A/CA. However, in contrast to practice elsewhere, perioperative oral antibiotics with or without mechanical bowel preparation was not included in Swiss guidelines until recently and was to our knowledge only marginally rarely used in Switzerland during the time of this study. Accordingly, we do not believe that this factor had any important influence on our findings.

Third, unidentified confounding factors could have contributed to the choice of one antibiotic regimen rather than the other but, to our knowledge, the chosen regimen were consistently used by specific surgeons or hospitals, without changes based on patients’ or operations’ characteristics.

Fourth, lacking information about dosage of antibiotics and re-dosing intervals, we assumed that surgeons followed the national guidelines which specify both parameters for the two regimen, but we cannot exclude differences that may have biased our findings.

Lastly, only a subset of SSI were evaluated with intraoperative cultures and susceptibility to antibiotics was not available in a standardized way. Therefore, unrecognized differences in microbiological flora between the two patient groups may have influenced our results. However, the size of the cohort, including patients from all the Swiss regions, and the exclusion of patients who were already on antibiotics at the time of surgery should have mitigated this potential confounding factor.

## Conclusion

Our large multicentric observational study showed a clear benefit of cefuroxime + metronidazole over amoxicillin/clavulanic acid for systemic perioperative antibiotic prophylaxis in colorectal surgery, with and without adjustment for potential confounders and despite a better activity of the latter against enterococci. Therefore, A/CA should not be a preferred choice for antibiotic prophylaxis in colorectal surgery but local antimicrobial resistance patterns of the main pathogens should also be taken into consideration for that choice. In addition, the high colorectal SSI rates observed in this study argue for additional preventive efforts besides antibiotic prophylaxis, including a better implementation of evidence-based measures already in place.

### Supplementary Information

**Additional file 1. Figure**: Flow chart. **Tables**: Risk factors for superficial (A), deep incisional (B), and organ-space SSI (C) A. Superficial incicisional infections.

## Data Availability

Data supporting reported results will be available upon request for the peer-review process.
