# Supplementary material for: Association of antimicrobial perioperative prophylaxis with cefuroxime plus metronidazole or amoxicillin/clavulanic acid and surgical site infections in colorectal surgery
Source: Antimicrob Resist Infect Control. 2023 Sep 19;12:105. doi: 10.1186/s13756-023-01307-y (PMC10510121; doi:10.1186/s13756-023-01307-y)
Supplement: Supplementary file 1 — Additional file 1. Figure: Flow chart. Tables: Risk factors for superficial (A), deep incisional (B), and organ-space SSI (C) A. Superficial incicisional infections. [file 13756_2023_1307_MOESM1_ESM.docx]

**SUPPLEMENTARY MATERIAL**

Figure: Flow chart

27,922 patients

55,668 colorectal surgeries 2009-2018

Excluded:

- Contamination class III, IV (n=18,128)
- Missing data (n=1,264)
- Other SAP than C+M or A/CA (n=7,612)
- Already on antibiotic therapy before the operation (n=742)

26,087 C+M

1,835 A/CA

Propensity score matching

1,835 A/CA

1,835 C+M

SAP: antimicrobial prophylaxis; C+M: cefuroxime + metronidazole; A/CA : amoxicillin/clavulanic acid

Tables: Risk factors for superficial (A), deep incisional (B), and organ-space SSI (C)

A. Superficial incicisional infections

| Risk factor | Crude odds ratio [95% CI] | P value | Adjusted odds ratio [95% CI] | P value |
| --- | --- | --- | --- | --- |
| Amoxicillin/clavulanic acid | 1.89 [1.46, 2.45] | <0.001 | 1.90 [1.51, 2.39] | <0.001 |
| Age (10-year steps) | 1.06 [1.01, 1.12] | 0.03 | ns | ns |
| Female sex | 0.80 [0.70, 0.92] | 0.002 | 0.86 [0.75, 0.99] | 0.04 |
| ASA score > 2 | 1.78 [1.61, 1.96] | <0.001 | 1.41 [1.26, 1.59] | <0.001 |
| Operation duration (per 30 minutes) | 1.05 [1.02, 1.08] | 0.001 | 1.06 [1.03, 1.08] | <0.001 |
| Antibiotic prophylaxis between 60 to 30 minutes before incision* | 0.96 [0.89, 1.04] | 0.3 | ns | ns |
| Duration of surgery >75^th^ percentile) | 1.18 [0.97, 1.43] | 0.1 | ns | ns |
| Elective surgery | 0.49 [0.40, 0.60] | <0.001 | 0.66 [0.53, 0.81] | <0.001 |
| Laparoscopic use | 0.43 [0.38, 0.50] | <0.001 | 0.51 [0.44, 0.59] | <0.001 |
| Rectal surgery | 0.83 [0.56, 1.22] | 0.3 | ns | ns |
| Hospital size (N beds)   - 200-499 - 500+ | 1.27 [0.88, 1.85]  1.37 [1.00, 1.87] | 0.2  0.05 | ns  ns | ns  ns |
| Re-operation** | 2.94 [2.34, 3.68] | <0.001 | 2.48 [1.98, 3.10] | <0.001 |

*For the first antibiotic administered (if in combination).

**For a non-infectious complication during the follow-up period.

Abbreviations: CI = confidence interval; ASA = American Society of Anesthesiology; ns = not significant (not retained in the multivariable model).

B. Deep incisional SSI

| Risk factor | Crude odds ratio [95% CI] | p-value | Adjusted odds ratio [95% CI] | p-value |
| --- | --- | --- | --- | --- |
| Amoxicillin/clavulanic acid | 1.44 [0.84, 2.47] | 0.2 | ns | ns |
| Age (10-year steps) | 1.02 [0.96, 1.09] | 0.4 | ns | ns |
| Female sex | 0.75 [0.62, 0.91] | 0.004 | ns | ns |
| ASA>2 | 1.99 [1.66, 2.38] | <0.001 | 1.53 [1.25, 1.86] | <0.001 |
| Operation duration (per 30 minutes) | 1.11 [1.07, 1.15] | 0.001 | 1.10 [1.07, 1.14] | <0.001 |
| Antibiotic prophylaxis between 60 to 30 minutes before incision | 0.93 [0.87, 0.99] | 0.02 | 0.93 [0.88, 0.98] | 0.005 |
| Duration of surgery >75^th^ percentile | 1.63 [1.33, 1.98] | <0.001 | ns | ns |
| Elective surgery | 0.50 [0.38, 0.66] | <0.001 | 0.74 [0.54, 1.02] | 0.07 |
| Laparoscope use | 0.35 [0.28, 0.45] | <0.001 | 0.43 [0.34, 0.55] | <0.001 |
| Rectal surgery | 1.33 [0.99, 1.79] | 0.06 | ns | ns |
| Hospital size (N beds)   - 200-499 - 500+ | 1.46 [1.04, 2.06]  1.69 [1.12, 2.56] | 0.03  0.01 | 1.37 [1.00, 1.89]  ns | 0.05  ns |
| Re-operation | 5.81 [4.06, 8.31] | <0.001 | 4.83 [3.26, 7.14] | <0.001 |

C. Organ space SSI

| Risk factor | Crude odds ratio [95% CI] | P-value | Adjusted odds ratio [95% CI] | P-value |
| --- | --- | --- | --- | --- |
| Amoxicillin/clavulanic acid | 1.23 [0.99, 1.53] | 0.06 | 1.27 [1.01, 1.60] | 0.05 |
| Age (10-year steps) | 1.01 [0.98, 1.05] | 0.5 | ns | ns |
| Female sex | 0.60 [0.54, 0.66] | <0.001 | 0.66 [0.60, 0.73] | <0.001 |
| ASA>2 | 1.57 [1.41, 1.73] | <0.001 | 1.33 [1.19, 1.50] | <0.001 |
| Operation duration (per 30 minutes) | 1.09 [1.07, 1.11] | 0.001 | 1.08 [1.06, 1.10] | <0.001 |
| Antibiotic prophylaxis between 60 to 30 minutes before incision | 0.94 [0.89, 0.99] | 0.02 | 0.95 [0.91, 1.00] | 0.04 |
| Long duration of surgery (>75^th^ percentile) | 1.45 [1.30, 1.62] | <0.001 | ns | ns |
| Elective surgery | 0.68 [0.59, 0.78] | <0.001 | 0.84 [0.73, 0.97] | 0.02 |
| Laparoscopic surgery | 0.63 [0.54, 0.73] | <0.001 | 0.72 [0.62, 0.84] | <0.001 |
| Rectal surgery | 1.18 [0.73, 1.91] | 0.5 | ns | ns |
| Hospital size (in number of beds)   - 200-499 - 500+ | 0.99 [0.72, 1.35]  1.20 [0.96, 1.51] | 0.9  0.1 | ns  ns | ns  ns |
| Re-operation | 5.77 [4.25, 7.83] | <0.001 | 5.22 [3.81, 7.15] | <0.001 |
